# 全水相微流控系统一步制备球丝异质载体用作细胞三维培养

**DOI:** 10.3724/SP.J.1123.2023.06008

**Published:** 2023-09-08

**Authors:** Mengqian ZHAO, Haitao LIU, Xu ZHANG, Zhongqiao GAN, Jianhua QIN

**Affiliations:** 1.中国科学院大连化学物理研究所, 辽宁 大连 116023; 1. Dalian Institute of Chemical Physics, Chinese Academy of Sciences, Dalian 116023, China; 2.中国科学院大学, 北京 100049; 2. University of Chinese Academy of Sciences, Beijing 100049, China

**Keywords:** 全水相微流控系统, 球丝异质载体, 细胞载体, all-aqueous microfluidic system, droplet-filled hydrogel microfibers, cellular carriers

## Abstract

水凝胶微丝是一种在生物医学领域备受关注的支架材料,具有良好的生物相容性、可调的力学性能和较大的比表面积。然而,在绿色环境下制备高细胞负载能力和多组分载荷的异质微丝仍然面临挑战。为了克服这一问题,本研究建立了一种基于气动泵阀辅助的全水相微流控系统,该系统能够实现具有球丝异质形态和先进功能的水凝胶微载体的一步制备。在这个系统中,利用右旋糖酐和聚乙二醇两相的自发相分离,形成液滴,并利用海藻酸钠和氯化钙的离子交联固化,形成水凝胶。通过调整内相、中间相和外相的流速,可以灵活控制液滴的大小、液滴之间的间距和微丝的宽度。得到的水凝胶微丝具有等距离排列的液滴,呈现出球丝异质的形态。进一步的实验结果表明,这种水凝胶微丝载体可以用于高通量原位生成三维细胞球。生成的细胞球表现出良好的细胞存活率和药物测试功能。这说明该载体在细胞培养方面具有潜在的应用前景。该全水相微流控系统具有高效、精确和可控的特点,为水凝胶微丝的制备提供了新的方法。这一技术的开发为进一步开展生物医学研究和应用提供了有力支持,也为制备用于材料科学、组织工程和药物测试的多功能水凝胶微丝提供了新途径。

## Abstract (en)

Hydrogel microfibers, which are characterized by flexible mechanical properties, a uniform spatial distribution, large surface areas, and excellent biocompatibility, hold great potential for various biomedical applications. However, the fabrication of heterogeneous hydrogel microfibers with high cell-loading capacity and the ability to carry multiple components via an environmentally friendly method remains challenging. In this study, we developed a novel pneumatic pump-assisted all-aqueous microfluidic system that enables the one-step fabrication of all-aqueous droplet-filled hydrogel microfibers with unique morphologies and adjustable configurations. By designing a pump-valve cycling system and selecting two immiscible fluids with stable water interfaces (dextran and polyethylene glycol), we successfully fabricated alginate microfibers with equidistantly arranged droplets through the ionotropic gelation reaction between sodium alginate and calcium chloride. The droplet size, interdroplet spacing, and microfiber dimensions could be flexibly controlled by adjusting the flow rates of the inner-phase, middle-phase, and outer-phase inlets. The results showed that the system enabled the high-throughput in situ formation of functional three-dimensional cell spheroids. The generated cell spheroids exhibited excellent cell viability and drug-testing functionality, indicating their potential applications in cell cultures. The developed technique offers strong support for future biomedical research and applications, and provides a new approach for the preparation of multifunctional hydrogel microfibers for materials science, tissue engineering, and drug testing.

水凝胶微丝因具有较大的比表面积、优异的生物降解性和可控的力学性能,在材料科学、细胞生物学和组织工程领域展现出巨大的潜力^[1⇓⇓⇓⇓-6]^,并被广泛应用于移植载体^[7,8]^、药物传递^[9,10]^和水凝胶传感器^[11,12]^等。制备水凝胶微丝的方法主要包括静电纺丝^[13,14]^、湿纺丝^[15,16]^、自组装^[17⇓⇓-20]^、生物打印^[19⇓-21]^和微流控技术^[22,23]^等。近年来,微流控技术凭借其芯片设计的灵活性和流体控制的准确性,在制备具有不同结构、成分和功能的水凝胶微丝方面获得了广泛的关注^[24]^。在微流控纺丝过程中,稳定的液体喷流通常会被鞘流包围,并经过物理或化学反应生成固体微丝^[25⇓-27]^。根据形态特征,可将微流控纺丝的产物分为中空管状微丝^[28,29]^、凹槽微丝^[30,31]^、螺旋微丝^[32,33]^和球丝^[34]^等。其中,球丝包含液滴单元,具有可调的液滴大小和间距、显示出更强的多组分载荷能力和促进多功能细胞团簇形成等巨大优势。

近年来,研究人员多利用油-水双相体系,在连续乳化步骤中制备含油滴的水凝胶球丝^[35⇓-37]^。这些研究大大提升了水凝胶微丝的结构复杂性和应用功能性,并为新型微丝的设计和制备提供了很好的范例。然而,上述方法存在明显的局限性,即微丝中存在油相,只适于对疏水性物质进行负载。若作为细胞载体,可能会影响内部细胞或组织的存活率及其功能,且液滴中的油相可能会阻碍微丝内外的物质交换^[38⇓-40]^。虽然也有研究利用双水相体系成功制备了同时填充水滴和油滴的水凝胶微丝,但这种方法不可避免地引入了油相,制备好的载体仍无法避免清洗除油等复杂的后处理过程^[40]^。因此,如何在无油系统中灵活地制备具有高生物相容性和良好功能性的球丝异质载体在生物医学研究中具有重要价值。

为应对上述挑战,本文提出了一种基于全水相微流控系统的微丝制备新方法,可通过在海藻酸钙微丝中排列液滴,生成包含水滴的球丝异质载体。将右旋糖酐(DEX)、含海藻酸钠(NaA)的聚乙二醇(PEG)和氯化钙(CaCl_2_)溶液分别设置为内相、中间相和外相流体,基于DEX与PEG间的相分离作用,生成全水液滴,并依靠NaA和CaCl_2_之间的离子交联反应在液滴外部形成水凝胶微丝。同时,通过调整三相流体的流速,可以灵活控制全水球丝的形态,包括液滴的大小、间距和微丝的宽度。为了验证该微丝用于细胞三维(3D)培养的可行性,我们在微丝的液滴中负载了人非小细胞肺癌细胞(A549)。经过一周的培养,该细胞可高通量原位生成3D细胞球,显示出良好的细胞存活率和药物测试功能。本研究建立的全水球丝异质结构制备方法具有可控性和灵活性,为进一步开展生物医学应用和研究提供了有力支持,也为制备用于材料科学、组织工程、药物测试和体内移植的多功能水凝胶载体提供了新途径。

## 1 实验部分

### 1.1 仪器和试剂

实验仪器:Spin master 51型匀胶机(上海凯美特功能陶瓷技术有限公司), UV-KUB 2型紫外曝光机(Kloe,法国), BP-2B型烘胶机(北京创世威纳公司), TS-PL30MA型氧等离子清洗器(深圳市东信高科自动化设备有限公司),离子溅射仪(中科科仪技术发展有限公司),aqueous 11 Picro Plus型注射泵(Harvard,美国), TM3000型扫描电子显微镜(Hitachi,日本), FV3000型激光共聚焦显微镜(Olympus,日本), LeicaIX 71型倒置荧光显微镜(Leica,日本), SCIENTZ-10ND冷冻干燥机(宁波新芝公司), Steri-Cycle CO_2_细胞培养箱(Thermo,美国), CYT5MFV型酶标仪(BioTek,美国)。

实验试剂与材料:Sylgard 184 PDMS单体及引发剂(Dow Corning,美国), SU-8 3035光刻胶(Microchem,美国), 3英寸直径单面抛光单晶硅片(浙江立晶光电科技公司),乳酸乙酯、无水氯化钙和氯化钠(天津大茂化学试剂厂),右旋糖酐(500 kDa)、聚乙二醇(20 kDa)(阿拉丁试剂有限公司),海藻酸钠(55 cps,青岛海之林公司);所有试剂均为分析纯或以上纯度。人非小细胞肺癌细胞、高糖DMEM培养基、胎牛血清、胰蛋白酶和细胞死/活染色试剂盒(Gibco,美国),青-链霉素(上海碧云天公司),细胞计数试剂-8(CCK-8,大连美伦公司), 磷酸缓冲盐溶液(PBS, Thermo,美国),去离子水(杭州娃哈哈集团有限公司)。

### 1.2 微流控芯片设计与制作

采用多层软光刻技术进行微流控芯片制作。用匀胶机以1000 r/min的速度在硅片上甩光刻胶SU-8 3035,在95 ℃的烘胶台上进行20 min前烘;室温冷却后,使用第一层结构掩膜在紫外下曝光,随后置于烘胶台上后烘20 min;重复曝光和后烘步骤,进行第二层和第三层结构掩膜的曝光。再使用乳酸乙酯进行显影,并使用压缩空气吹干,于烘箱180 ℃静置1 h进行坚膜。所制备的SU-8模板具有3层结构,其中内相和气阀通道的高度为200 μm,中间相和外相通道的高度为325 μm和525 μm。内、中、外通道的宽度分别为65、450和1500 μm。将PDMS单体和引发剂以10∶1(v/v)完全混合均匀,浇筑在模板上,除气泡后,在80 ℃下加热成型。分离PDMS和SU-8模板,将两块PDMS置于等离子体中处理(250 W, 30 s)后,上下对叠使结构重合,进行芯片的封接。

### 1.3 球丝的制备和表征

以DEX和PEG溶液为全水相体系,将以下试剂注入微流控芯片:(1)15%(质量分数,下同)DEX溶液注入内部流道,(2)包含17% PEG和1% NaA的混合溶液注入中间流道,(3)包含17% PEG和4% CaCl_2_的混合溶液注入外部流道。使用含有17% PEG和4% CaCl_2_的混合溶液样品池来收集球丝。

使用光学显微镜和ImageJ对球丝进行拍照表征并对液滴的直径、液滴间距和微丝宽度进行尺寸统计。为了进一步表征球丝的结构,我们在中间流道中加入了荧光素5-异硫氰酸酯标记的NaA(FITC-NaA,绿色,最终质量浓度:1 mg/mL),并使用荧光显微镜进行拍照表征。将收集的球丝进行乙醇梯度脱水,置于冻干机中冻干72 h,取出进行离子溅射处理,并使用扫描电镜拍照表征。

### 1.4 肿瘤细胞的培养和负载

A549细胞培养在高糖DMEM培养基中,培养环境为含5% CO_2_的37 ℃培养箱,细胞每2天按照1∶3比例传代一次。当细胞在培养皿中生长至80%以上覆盖率时,用胰蛋白酶在37 ℃条件下消化2 min,然后置于离心管中收集(800 r/min, 3 min)细胞;弃掉上清液,加入15%DEX溶液重悬,细胞密度为1×10^7^个/mL,通入内部流道,进行细胞的原位负载,以上实验中所用芯片和可耐高温的试剂均经过高压灭菌处理,且实验过程在超净台中完成。将收集到的细胞负载微丝转移至含有高糖DMEM培养基的孔板中,在含5% CO_2_的37 ℃培养箱中培养,每2天弃去上层培养基,更换新培养基。

### 1.5 肿瘤细胞抗癌药物测试

将A549细胞接种在96孔板中,进行2D培养,同时将收集的负载细胞的球丝定量收集在96孔板中培养,培养至第4天。对2D培养和负载于球丝的A549施加0.1、1、10、100和1000 μmol/L的顺铂,进行24 h的药物刺激,然后用CCK-8测量细胞活性,用死/活染色试剂进行染色,并使用共聚焦拍照表征。

## 2 结果与讨论

### 2.1 全水球丝制备系统及操控原理

为了灵活可控地制备全水球丝,我们设计并建立了一种全水相微流控系统,将液滴微流控技术与湿法纺丝方法相结合。如图1a所示,微流控装置包括一系列关键单元,包括流体进口单元、液滴生成单元、微丝生成单元和收集单元。在该系统中,以DEX和PEG的水溶液作为全水体系制备球丝。具体而言,分别通过内相进口、中间相进口和外相进口引入DEX溶液、含有NaA的PEG溶液和含有CaCl_2_的PEG溶液。由于PEG比DEX更疏水^[41]^,因此中间相的PEG与PDMS通道有更好的亲和力,在细胞封装过程中具有更好的包覆效果。液滴生成单元采用“十”字交汇几何形状,用于在两种不相溶的液体(DEX和PEG)之间形成剪切力,生成液滴。同时,位于内相通道旁边的单层膜气阀可在外部电磁阀的作用下周期性改变内相流体的通断,从而增加液滴生成过程的可操作性以及微丝中液滴排列的可控性(图1b)。气动阀的存在也使得生成具有预定义液滴阵列的球丝成为可能。经过气阀挤压后形成大小均一的液滴包裹于NaA预聚物溶液中,通过与外相流体中CaCl_2_在通道汇集口交汇后,进入微丝生成单元,经离子交联快速固化形成海藻酸盐水凝胶微丝,液滴原位均匀分散于微丝之中,并且流入包含PEG溶液的收集池中(交联原理如图1c所示)。全水球丝可以作为具有多个隔室的均匀载体,用于携带各种化学组分或细胞球体,并且可以实现高通量制备,该微丝也可以轻松组装成更复杂的结构^[29]^。这些微丝可以用于重建具有丝状基质的功能组织,模拟肌肉组织、血管或神经网络^[8,41,42]^。此外,在糖尿病治疗方面,球丝可以在纵向上扩大胰岛的培养量,便于操作、植入和取出。这个特性可以最大程度地减小移植的安全风险^[43⇓-45]^。

**图 1:**
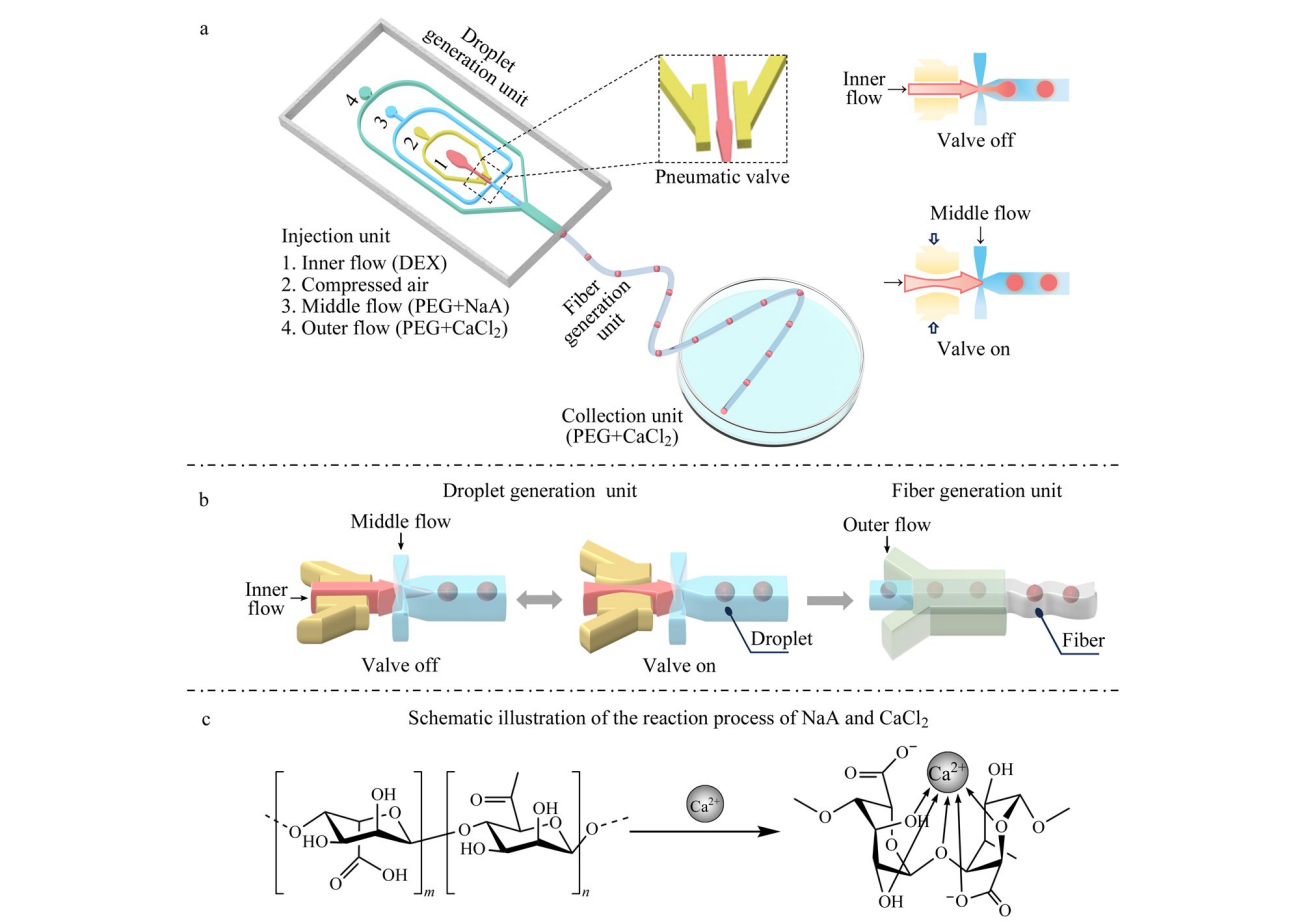
用于全水球丝水凝胶微丝制备的微流控系统示意图

### 2.2 全水球丝的可视化

全水相系统制备的水凝胶微丝中的水相液滴能够提升该方法在细胞生物学应用中的优势。将内相、中间相和外相的流速分别固定为0.6、5.5和300 μL/min,进行球丝的收集,并使用显微镜拍照观察液滴生成单元和微丝生成单元(图2a)。在气阀的作用下,内相和中间相流体经过“十”字通道后,稳定生成被NaA预聚体包裹的大小均一的液滴,再进一步进入外相汇集通道,与CaCl_2_反应生成海藻酸盐微丝。收集池中的微丝具有良好的力学性能,可以用镊子夹起(图2b i),液滴均匀地分布在水凝胶微丝中(图2b ii和2b iii)。为了进一步观察球丝的结构,我们将FITC标记的海藻酸钠作为中间相流体注入芯片中进行微丝制备,并利用共聚焦显微镜拍照表征(图2c)。结果显示,基于该方法生成的微丝是完整且连续的,其中包裹的液滴中并没有绿色荧光,说明中间相的NaA无法扩散到内相中,并且液滴和微丝之间的分隔十分显著。这些结果表明,全水球丝的建立有利于以可控的空间排列方式共负载多种成分,以满足多种潜在需求,并展现出灵活的功能性和可控的复杂性。随后,经乙醇梯度脱水和冷冻干燥处理,可在扫描电子显微镜(SEM)下对微丝的结构进行表征。如图2d i所示,全水球丝完整、光滑且连续,表明本研究方法可以有效降低球体损失的风险,从而为移植过程提供更多的可行性。此外,图2d ii显示该球丝在液滴之间的间隔区域明显收缩,并呈现出纺锤结构,表明微丝中包含的液滴为中空结构。图2d iii显示的是垂直于微丝轴向切割之后的截面图,可以更清晰地展示微丝中的空腔结构,这种具有连续含水相空腔的微丝,很难通过传统方法制备获得。这些结果也展示了球丝材料的高含水性,这是生物医学应用中的关键特性之一。

**图 2:**
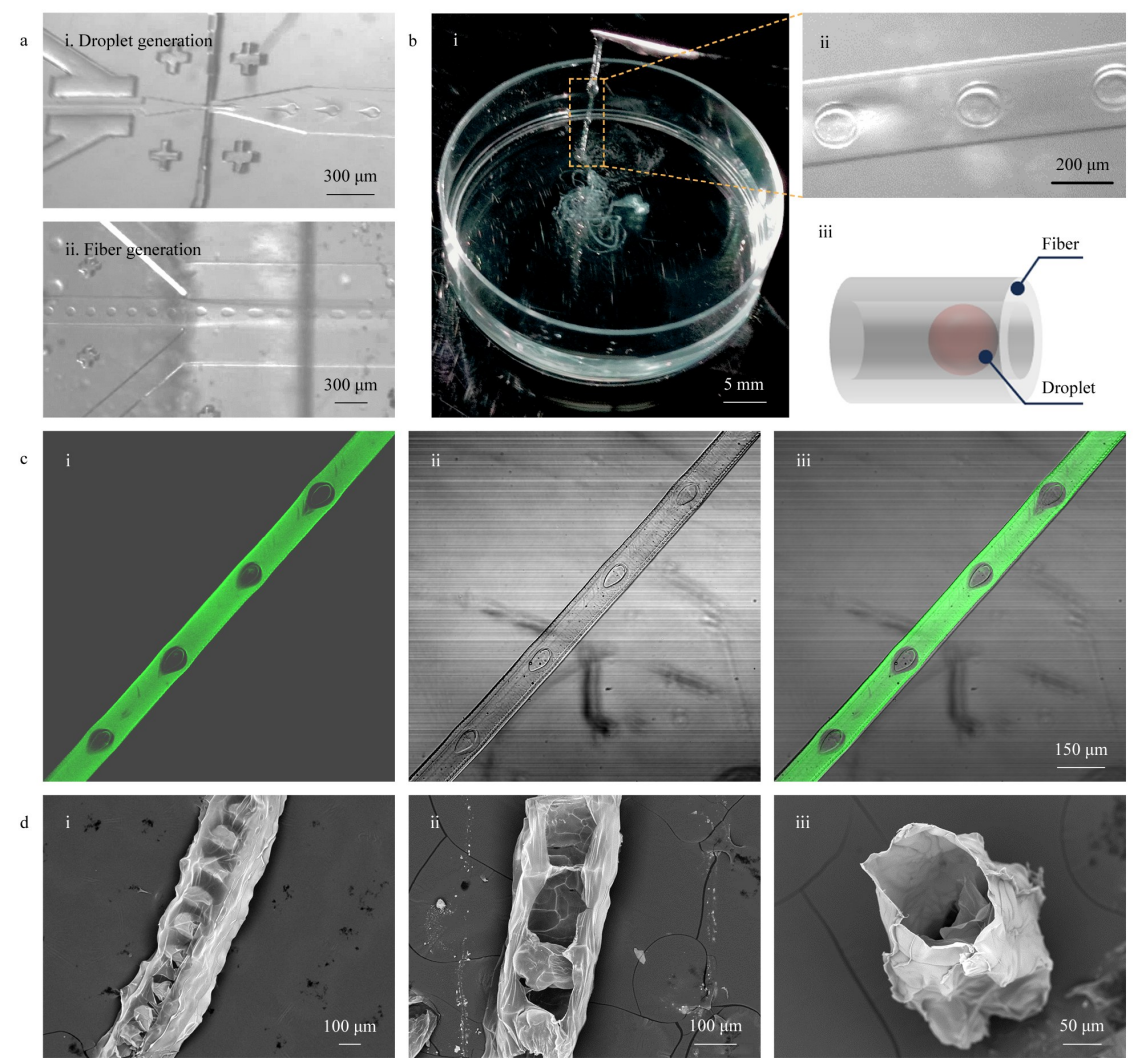
全水球丝制备以及形貌表征

### 2.3 多相流体流速对微丝形貌的影响

为研究球丝制备过程中的关键影响因素,我们系统地分析了在不同多相流体流速下液滴直径、液滴间距和微丝宽度的变化规律。在该研究过程中,阀门切换周期始终设定为0.4 s,外相流速固定为300 μL/min。首先,我们通过调节内相流速(0.2 ~1.0 μL/min)和中间相流速(2.5~10.5 μL/min)制备微丝,收集微丝并置于显微镜下拍照表征。结果表明,随着流体流速的改变,微丝的形貌也会发生变化,共收集到5种形貌的微丝,根据其中液滴形态的变化,可分为:紊乱形液滴微丝、圆球形液滴微丝、连接形液滴微丝、波浪形中空微丝、直线形中空微丝(图3)。统计表明,中间相流速与内相流速之比较小时,中间液滴会出现紊乱现象,无法形成有序排列的液滴,随着比值增加,两相流体趋向于稳定,可以形成较圆的液滴。但随着内相流体流速的进一步增加,即使在气阀的辅助作用下,中间相流体也无法将内相切断,因而会出现多个液滴连接在一起的现象。若内相流体流速继续增大,则无法形成液滴,此时将获得波浪形中空微丝和直线形中空微丝。

**图 3:**
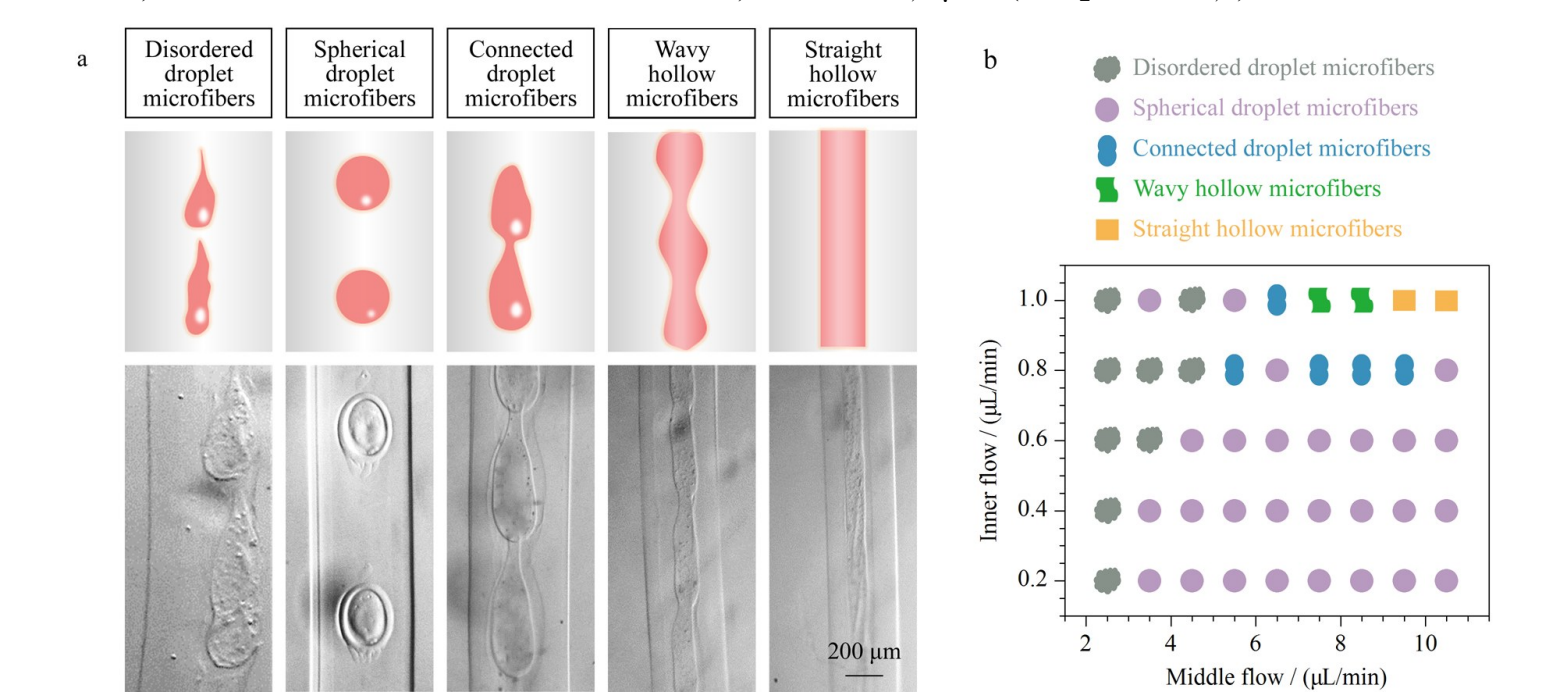
海藻酸盐微丝中不同形状的液滴包封分布图及表征图

随后,我们在可以稳定形成球丝的流速范围内,将中间相和外相的流速分别固定为5.5 μL/min和300 μL/min,观察了内相流速对微丝形貌的影响。如图4a所示,当内相流速从0.2 μL/min增加至0.8 μL/min时,内相流速与丝内液滴的直径呈正相关,液滴直径从(41.03±2.76) μm增加到(96.59±3.31) μm (***p<0.001),但内相流速几乎不影响液滴间距和微丝直径(*p*>0.05)。类似地,为了评估中间相的影响效应,我们将内相和外相的流速分别固定为0.6 μL/min和200 μL/min,然后调整中间相的流速(3.5~6.5 μL/min)。如图4b所示,随着中间相流速的增加,液滴直径小幅度增加,从(61.61±2.31) μm增加到(70.04±2.97) μm (***p<0.001),而液滴间距也发生变化,从(438.51±38.05) μm增加到(546.51±45.99) μm (***p<0.001),而微丝直径没有显著性变化(*p*>0.05)。

**图 4:**
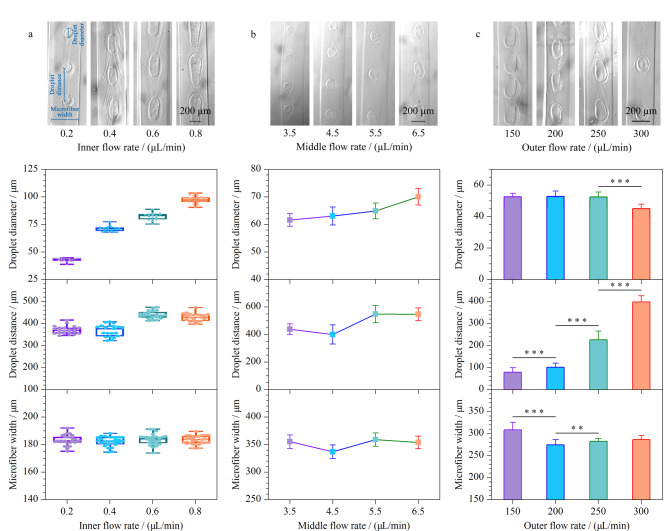
不同(a)内相、(b)中间相和(c)外相流速下球丝异质载体的明场图和对液滴直径、液滴间距和微丝宽度的统计图

图4c显示了外相流速对微丝制备的影响,此时内相和中间相的流速分别固定为0.6 μL/min和5.5 μL/min。在流速较小时,外相流速的增加(150~300 μL/min)未对微丝的形成产生明显影响。但外相流速在250 μL/min和300 μL/min两组间,液滴直径从(52.48±2.26) μm降低到(45.04±2.79) μm (***p<0.001),液滴间距在每一组相邻流速中均存在显著性增加,从(77.91±21.25) μm增加到(397.04±29.17) μm (***p<0.001);微丝直径则从(308.23±17.18) μm降低为(286.27±9.51) μm (***p<0.001),即在相同的内相和中间相流速下生成了更长且更细的丝。

上述结果表明,液滴的大小主要取决于内相的流速,而液滴之间的距离主要取决于中间相和外相的流速,微丝的大小则主要取决于外相的流速。因此,通过灵活改变流速,可以调控球丝的尺寸以及液滴的位置和大小。与通过油-水两相系统产生的水凝胶球丝不同,本研究所提出的全水微流控系统可以扩展水凝胶微丝的应用范围,例如在水相液滴中封装亲水性物质^[46,47]^。此外,这种方法有利于精确调控含有一系列水相液滴的水凝胶微丝,可以作为一种细胞友好的载体,或用于可控地封装具有特定含量的成分。同时,该全水球丝可以作为潜在的可植入物质传递载体,以满足生物和医学应用的不同需求^[44,45,48]^。

### 2.4 球丝生物相容性考察

为了验证球丝作为组织工程应用中三维载体的可行性和生物相容性,我们在微丝制备过程中将A549细胞负载其中。具体为:将接种于培养瓶中的A549细胞消化后进行离心,重悬于内相溶液中(DEX溶液,1×10^7^个/mL),并封装到CaA丝的液滴中(图5a)。在随后的细胞培养过程中,我们测量了微丝中细胞数量的分布,以及细胞的死活比例。结果表明,随着培养时间的增长,细胞在微丝中发生明显的聚集和增殖,并最终形成致密的3D肿瘤细胞球。凝胶中Ca^2+^和溶液中Na^+^发生离子交换,使凝胶中的 COO^-^基团增加,导致聚合物链松弛,进一步导致海藻酸盐水凝胶的溶胀和降解。随着时间的推移,藻酸盐和培养基中的Ca^2+^达到平衡状态,降解过程相应减慢。我们制备的负载细胞的海藻酸钙水凝胶微丝在高糖DMEM培养基中培养一周后,微丝以及所包裹的液滴形貌保持良好并未发生明显的塌缩和降解,可用镊子夹起进行染色和拍照处理,说明其作为细胞负载支架材料的可行性^[49,50]^。此外,本研究中收集的微丝可以缠绕形成水凝胶团块(图5b),从而实现方便灵活的取用和移动,并且一条完整的微丝中可以包含上百个细胞球,显示该系统在细胞负载中的高通量特点以及在移植应用中的潜在价值。通过对液滴中细胞量的测定,发现肿瘤细胞球的直径显著增加,从(76.12±7.45) μm增长至(117.79±2.98) μm。此外,死/活试剂染色结果表明,随着培养时间的增加,负载于液滴微丝中的细胞存活良好(绿色荧光为活细胞),并保持了良好的增殖率,这与明场下细胞的形态变化一致(图5c)。肿瘤细胞球中的活细胞比例在1周内稳定且高于90%(图5d),证明该材料及其制备过程均具有很好的生物相容性。

**图 5:**
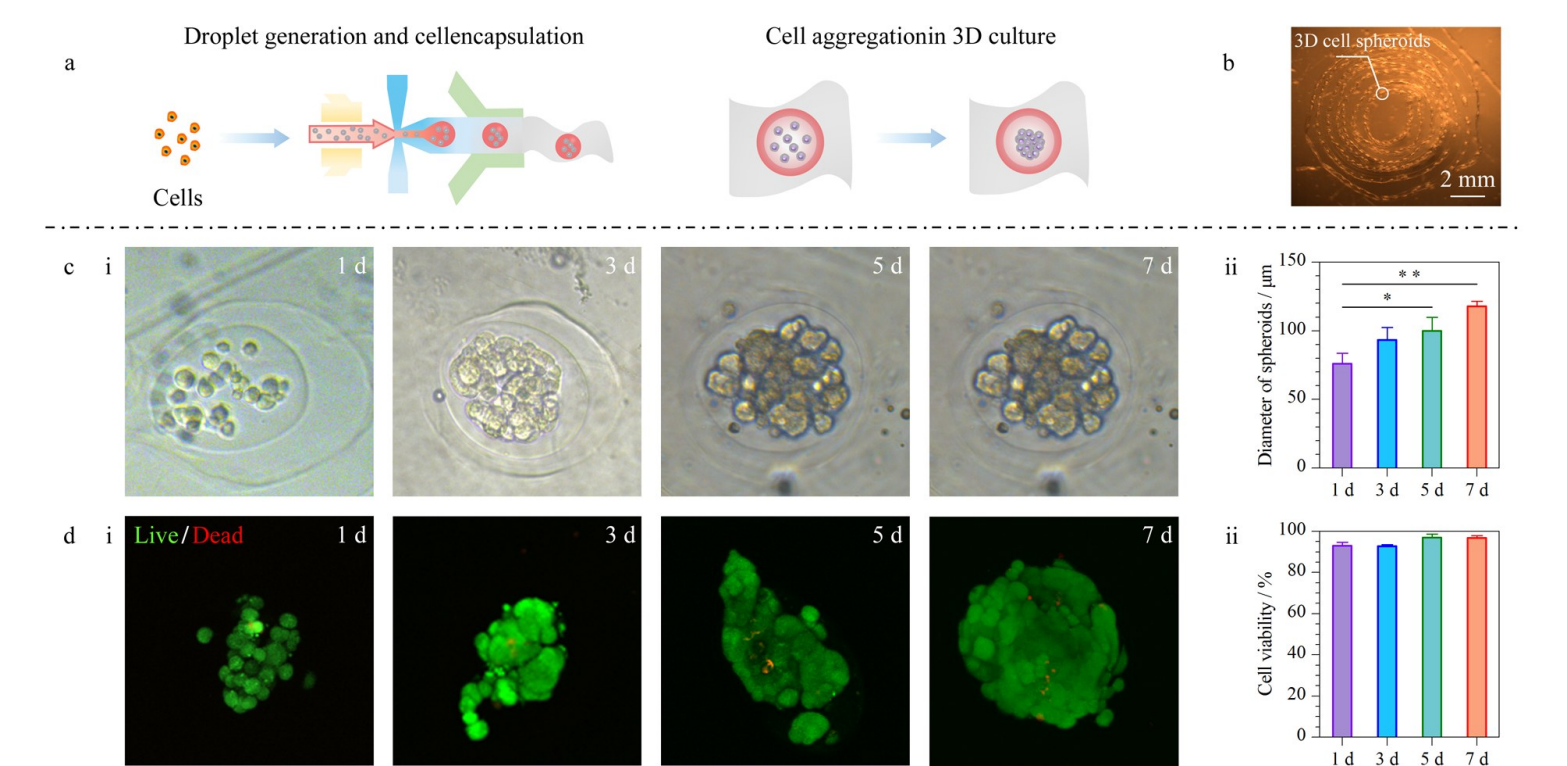
全水球丝中负载和形成3D细胞球

### 2.5 球丝负载细胞球药物测试功能考察

为了验证微丝中肿瘤细胞球的应用价值,我们对A549细胞进行了体外抗肿瘤药物测试。即在培养基中加入不同浓度(0.1、1、10、100和1000 μmol/L)的抗癌药物顺铂,作用24 h之后,测定细胞活性的变化。同时,我们将直接接种于96孔板中2D培养的A549细胞作为对照组,加入同样浓度的药物刺激相同时间后进行细胞活力测定(图6)。结果表明,2D培养的A549细胞在100 μmol/L顺铂刺激后,细胞发生部分脱落,并且死细胞比例增加,相对细胞活性降低至27.06%±2.01%;而浓度增至1000 μmol/L时,细胞几乎全部脱落,且细胞相对活性趋近于0。而负载于微丝中的A549细胞,在100 μmol/L顺铂刺激下,细胞相对活性降低至82.66%±0.86%;浓度增至1 mmol/L时,细胞相对活性降低至48.04%±4.78%。

**图 6:**
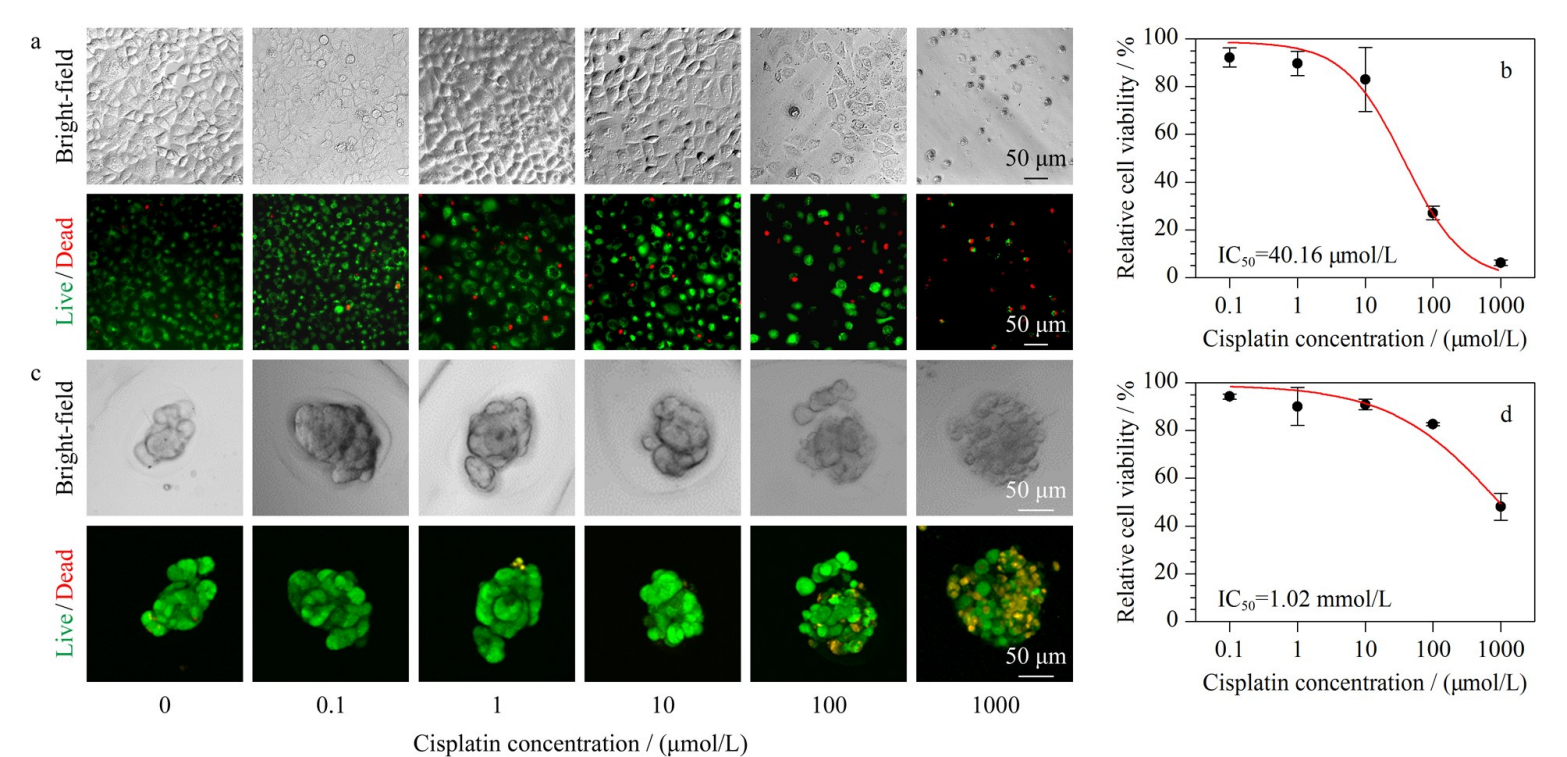
A549细胞球药物测试表征

随后,我们对两种培养方式的细胞进行剂量-反应曲线拟合,计算半抑制浓度(IC_50_)。结果显示,2D培养的A549的IC_50_为40.16 μmol/L,这与之前的报道^[51,52]^相接近。而负载于液滴微丝的3D肿瘤细胞球的IC_50_为1.02 mmol/L。在相同药物浓度下,3D细胞球比2D细胞球表现出更强的活力,表明3D细胞球具有更高的耐药水平。我们认为影响负载于微丝中的细胞球耐药性可能由不同的机制引起。其中一个原因是水凝胶微丝的存在影响了药物的扩散。海藻酸盐水凝胶作为一种多孔介质材料,对不同相对分子质量大小的物质具有不同的渗透性^[53,54]^。顺铂作为一种小分子药物(*M*_w_=300.05)可以缓慢扩散至海藻酸盐内部,相较于2D细胞直接暴露于药物中,微丝载体的存在减缓了细胞暴露于药物。另外在微丝的液滴中,形成了3D的致密肿瘤球,影响药物渗透进入细胞球内部,并且细胞球中的细胞密度、细胞外基质蛋白的表达也会影响扩散到球体中的药物功效^[55⇓⇓⇓⇓-60]^。这在一定程度上揭示了大多数2D细胞用于药物测试后,在体内应用中失败的原因,也证明了全水相系统制备的水凝胶微丝肿瘤模型在药物开发和筛选方面的巨大潜力。

## 3 结论

本研究建立了一种全新的全水相微流控纺丝系统,并灵活、可控地一步生成水凝胶球丝载体,避免了传统制备方法中油相的存在,提高了微丝体系的生物相容性。本研究中生成的全水球丝过程是完整、连续且可重复的。通过调节流速,可以灵活控制微丝中液滴的大小和排布。此外,利用此系统可高通量地获得肺肿瘤细胞球体外模型。负载球丝中的细胞可在一周的培养时间内保持良好的细胞活性,并表现出对抗癌药物顺铂的药物测试功能,显示出该肿瘤模型用于体外药物测试与筛选的价值。而该水凝胶微丝在结构与功能方面的特点也决定了其在材料科学和组织工程中的巨大应用潜力。
